# BODE-Index vs HADO-Score in Chronic Obstructive Pulmonary Disease: Which one to use in general practice?

**DOI:** 10.1186/1741-7015-8-28

**Published:** 2010-05-24

**Authors:** Cristóbal Esteban, José M Quintana, Javier Moraza, Myriam Aburto, Urko Aguirre, José I Aguirregomoscorta, Susana Aizpiri, Luis V Basualdo, Alberto Capelastegui

**Affiliations:** 1 Pneumology Department, Hospital Galdakao-Usansolo, Galdakao, Bizkaia, Spain; 2 Research Unit-CIBER Epidemiology and Public Health (CIBERESP), Hospital Galdakao-Usansolo, Galdakao, Bizkaia, Spain

## Abstract

**Background:**

Forced expiratory volume in one second (FEV_1_) is used to diagnose and establish a prognosis in chronic obstructive pulmonary disease (COPD). Using multi-dimensional scores improves this predictive capacity.Two instruments, the BODE-index (**B**ody mass index, **O**bstruction, **D**yspnea, **E**xercise capacity) and the HADO-score (**H**ealth, **A**ctivity, **D**yspnea, **O**bstruction), were compared in the prediction of mortality among COPD patients.

**Methods:**

This is a prospective longitudinal study. During one year (2003 to 2004), 543 consecutively COPD patients were recruited in five outpatient clinics and followed for three years. The endpoints were all-causes and respiratory mortality.

**Results:**

In the multivariate analysis of patients with FEV_1 _< 50%, no significant differences were observed in all-cause or respiratory mortality across HADO categories, while significant differences were observed between patients with a lower BODE (less severe disease) and those with a higher BODE (greater severity). Among patients with FEV_1 _≥ 50%, statistically significant differences were observed across HADO categories for all-cause and respiratory mortality, while differences were observed across BODE categories only in all-cause mortality.

**Conclusions:**

HADO-score and BODE-index were good predictors of all-cause and respiratory mortality in the entire cohort. In patients with severe COPD (FEV_1 _< 50%) the BODE index was a better predictor of mortality whereas in patients with mild or moderate COPD (FEV_1 _≥ 50%), the HADO-score was as good a predictor of respiratory mortality as the BODE-index. These differences suggest that the HADO-score and BODE-index could be used for different patient populations and at different healthcare levels, but can be used complementarily.

## Background

Forced expiratory volume in one second (FEV_1_) is an essential measure for diagnosing chronic obstructive pulmonary disease (COPD) and in establishing its severity and prognosis. Traditionally, the two most important prognostic factors in the COPD have been FEV_1 _and age [1,2]. Other variables, such as dyspnea [3], malnutrition [4], hospitalization related to COPD exacerbation [5], exercise capacity [6], physical activity [7], pulmonary hypertension [8], inspiratory capacity [9], lung density measurements by computed tomography [10], muscle mass [11], health-related quality of life [12], and other markers [13], have also proved to be individual, effective prognostic factors.

Simultaneously employing several variables improves the assessment of patients with COPD [11], and grouping these variables improves prognostic capacity compared with traditional classifications of COPD severity based exclusively on FEV_1_. In this regard, the BODE-index (**B**ody mass index, **O**bstruction, **D**yspnea, **E**xercise capacity) has proved to be a good predictor of respiratory and all-cause mortality [14]. The HADO-score (**H**ealth, **A**ctivity, **D**yspnea, **O**bstruction) is another multidimensional prognostic index that works well for predicting all-cause mortality [15].

The aim of this study was to compare the predictive capacity of the BODE-index and the HADO-score for all-cause and respiratory mortality in a large group of ambulatory patients with varying severity of COPD.

## Methods

Between January 2003 and January 2004, we recruited all patients previously diagnosed with COPD who regularly visited an outpatient clinic affiliated with the Hospital Galdakao-Usansolo, a teaching hospital with a catchment area of 300,000 inhabitants that could carry out the tests relevant to the study.

Consecutive patients were included in the study if they had been diagnosed with COPD at least six months previously and had been under treatment for at least six months. Patients had to be stable (no increase in respiratory symptoms or changes in treatment) for six weeks prior to inclusion. Other inclusion criteria were FEV_1 _< 80% of the predicted value, with an FEV_1_/FVC quotient <70% and a negative bronchodilation test with FEV_1 _change <15% of the baseline value or <200 ml. The functional parameters used were those obtained following bronchodilation. Patients were not eligible for the study if they had been diagnosed with asthma, had extensive pulmonary tuberculosis or neoplastic processes, were suffering from psychiatric or neurological problems that might prevent effective collaboration, or had hearing or other problems that impeded accurate communication.

The study was approved by the research committee of the Hospital Galdakao-Usansolo. Patients provided informed consent to take part in the study. All patients were followed for three years.

The BODE-index is made up of four variables (body mass index, FEV_1_, dyspnea, and a six-minute walking test). Its score ranges from 0 to 10 points in such a way that the higher the score the higher the severity [14]. The HADO-score is made up of four variables (the answer to a single, general, self-rated health question; self-rated level of physical activity; dyspnea; and FEV_1_). Its score ranges from 0 to 12, with a lower score indicating a higher severity and therefore a worse prognosis [15] (Table 1).

**Table 1:** Variables and point values used in BODE-index and HADO score

BODE-index	value	HADO-score	value
**FEV_1_%**		**FEV_1_%**	
≤65	0	≤65	3
50 to 64	1	50 to 64	2
36 to 49	2	36 to 49	1
≤35	3	≤35	0
**Dyspnea scale**		**Dyspnea scale**	
0 to 1	0	1	3
2	1	2	2
3	2	3	1
4	3	4 to 5	0
**6 MWT (m)**		**Physical Activity**	
≥350	0	3	3
250 to 349	1	2	2
150 to 249	2	1	1
≤149	3	0	0
**BMI**		**Health status**	
>21	0	very good/excelent	3
≤21	1	good	2
		fair	1
		bad	0
			

- FEV1% = forced expiratory volume in one second (% of predicted). Based on the stages of the American Thoracic Society.

- Dyspnea scale. ^16,17^

- Physical Activity: 0) 'doesn't leave the house, life is limited to the bed or armchair, or to doing some domestic chores', or 'leaves the house, but walks less than 100 meters'; 1) 'leaves the house and walks a few hundred meters, runs errands, but does not walk regularly'; 2) 'engages in physical activity in the vegetable garden', or 'takes walks for up to 8 kms, no less than five days a week'; and 3) 'takes walks regularly for longer than 8 kms, no less than five days a week', or 'practices sports'

- 6MWT = distance in meters walked in six minutes.

Patients were asked about their level of dyspnea using a five-degree modified Medical Research Council scale [16] and the scale adapted from Fletcher [17]: Degree 1, 'dyspnea only with intense and strenuous exercise'; Degree 2, 'capable of walking at the same pace as other people my age on the level'; Degree 3, 'capable of walking on the level at my own speed without dyspnea, but incapable of walking at the same pace as people my age'; Degree 4, 'dyspnea after walking slowly for 100 meters'; and Degree 5, 'dyspnea when resting or after slight effort such as getting dressed'.

General health was assessed by means of the question, 'In general, would you say your health is...'. The five possible answers were *poor, fair, good, very good*, and *excellent*.

Body mass index (BMI) was calculated by dividing weight in kilograms by the square of height in meters.

The six-minute walking test (6MWT) was carried out following American Thoracic Society (ATS) rules [18]. The best result was selected from two tests conducted with a 30-minute break between them.

Levels of physical activity were assessed on a scale from 0 to 3: 0) 'doesn't leave the house, life is limited to the bed or armchair, or to doing some domestic chores', or 'leaves the house, but walks less than 100 meters'; 1) 'leaves the house and walks a few hundred meters, runs errands, but does not walk regularly'; 2) 'engages in physical activity in the vegetable garden', or 'takes walks for up to 8 kms, no less than five days a week'; and 3) 'takes walks regularly for longer than 8 kms, no less than five days a week', or 'practices sports'.

Spirometry was conducted following criteria from Spanish Pneumology and Thoracic Surgery Society (SEPAR) [19], with a Master Scope-PC spirometer (Erich Jaeger GmbH & Co, KG, Wuerzburg, Germany). Theoretical values were those prescribed by the European Community for Steel and Coal [20].

We also measured the number of pack-years of cigarette smoking, established as the product of years as a smoker and the average number of cigarettes smoked per day, divided by 20.

Comorbidities were determined by reviewing the patients' clinical histories, applying the Charlson index [21]. This index has been developed to predict the mortality of patients with chronic disease. It generates a score that is proportional to the disease-related risk of death.

### Main outcomes

Vital status was initially determined by telephone calls made to patients or their next of kin. All reported deaths and dates of deaths were confirmed by reviewing medical reports, by examination of the hospital database and public death registries, or both. Deaths were considered confirmed if the record matched the subject on name, sex, and date of birth. In all cases, the cause of death was based on the hospital reports and public death registries. In cases in which death occurred out of the hospital, the researchers surveyed relatives and the primary care doctor. If the cause of death still remained unclear, a committee formed by some authors decided what the mortality was caused by.

### Statistical analysis

We present mean and standard deviations for continuous variables and frequencies and percentages for categorical variables. We used the Chi-square and Fisher's exact tests to evaluate associations among categorical variables.

Logistic regression models were used to study the ability of the BODE-index and HADO-score on vital status. Both all-cause and respiratory-related mortalities were evaluated. Additional analyses were performed using a FEV_1 _cut-off point of 50% to categorize patients as severe versus moderate/mild severity.

We performed a multivariate analysis, adjusting by age, cigarette packs/year, number of hospitalizations in the previous two years, and comorbidities measured by Charlson comorbidity index.

We computed the C statistic using logistic regression models to study the final predictive ability of the model. In these analyses, the C statistic is a mathematical function of the sensitivity and specificity of the score in classifying patients by means of a logistic regression model as either dying or surviving. The null value for the C statistic is 0.5, with a maximum value of 1.0 (higher values indicate better health).

All effects were considered significant at *P *< 0.05 unless otherwise noted. All statistical analyses were performed using SAS for Windows statistical software, version 8.2. (SAS Institute, Inc., Carey, NC, USA).

## Results

From a total of 808 consecutive COPD patients seen in one of the clinics, 265 were excluded from the study: 48 of these were not able to carry out the 6MWT, 63 had neoplasias (14 with pulmonary neoplasias), 30 had extensive bronchiectasis, 15 had fibrothorax, 18 had silicosis, 14 had extensive sequelae of pulmonary tuberculosis, 12 could not complete valid spirometry, 12 had senile dementia or Alzheimer's disease, 6 had a psychiatric pathology, 25 had miscellaneous other pulmonary and extrapulmonary reasons, and 22 refused to take part. This left 543 eligible patients; their characteristics are listed in Table 2. The sample was made up almost exclusively of men (96%). Patients had COPD of moderate severity (mean FEV_1_%, 55%) with a Charlson index of 2.4; 36% of the patients were admitted to the hospital at least once in the two years before being included in the study. Seventy-one patients (13.1%) died during the three-year follow-up period, primarily due to respiratory (45.1%), cardiovascular (18.3%), and cancer (12.7%) related conditions. The patients who died during follow up were older, had a significantly higher level of dyspnea, lower FEV_1_, lower exercise capacity in the 6MWT, and had been admitted to the hospital more often in the two years before the study began than the survivors. All the variables that made up the BODE-index and the HADO-score showed statistical differences between those patients still alive or dead at three years. In addition, their scores in the BODE-index and HADO-score showed significantly greater disease severity.

**Table 2:** Baseline characteristics of the study participants and characteristics by vital status after three years of follow up

	All Patients (n = 543)	Alive (n = 472)	Dead (n = 71)	*P*-Value
Age, yr	68.3 ± 8.3	67.8 ± 8.4	72.1 ± 6.5	<.001
Men	522 (96.1%)	452 (95.76%)	70 (98.59%)	0.34
FEV_1_, L	1.46 ± 0.44 L	1.50 ± 0.44	1.20 ± 0.36	<.001
FEV_1%_	55.0 ± 13.3	55.9 ± 13	49 ± 14	<.001
FEV_1_/VC	54.5 ± 9.32	54.7 ± 9.2	53.1 ± 10.5	0.16
Dyspnea scale				<.001
1	69 (12.7%)	62 (13.1%)	7 (9.9%)	
2	264 (48.6%)	245 (51.9%)	19 (26.8%)	
3	166 (30.6%)	136 (28.8%)	30 (42.3%)	
4-5	44 (8.1%)	29 (6.1%)	15 (21.1%)	
Distance walked in six minutes (m)	408.9 ± 92.4	420.1 ± 86.5	334 ± 96.6	<.001
Charlson index	2.4 ± 1.4	2.3 ± 1.3	3.3 ± 1.6	<.001
BMI	28.3 ± 4.4	28.4 ± 4.4	27.4 ± 4.8	0.09
Smoking habit				0.84
Current smokers	114 (20.9%)	101 (24.1%)	13 (18.3%)	
Ex-smokers	414 (76.2%)	358 (75.8%)	56 (78.9%)	
Never smoked	15 (2.8%)	13 (2.7%)	2 (2.8%)	
Mean pack/year, SD	48.2 ± 26.5	47.7 ± 26.3	51.1 ± 28	0.32
BODE index	2.8 ± 1.8	2.6 ± 1.6	4.2 ± 1.9	<.001
HADO score	6.8 ± 2.1	7 ± 2	5.6 ± 2.1	<.001
Previous admissions				<.001
0	402 (74%)	370 (78.4%)	32 (45.1%)	
1	88 (16.2%)	65 (13.8%)	23 (32.4%)	
≥2	53 (9.8%)	37 (7.8%)	16 (22.5%)	
				
Distance walked in 6 min (m)				<0.001
≤ 149	5 (0.92%)	4 (0.85%)	1 (1.41%)	
150 to 249	27 (4.97%)	14 (2.97%)	13 (18.31%)	
250 to 349	91 (16.76%)	66 (13.98%)	25 (35.21%)	
≥ 350	420 (77.35%)	388 (82.20%)	32 (45.07%)	
Physical activity				<0.001
0	28 (5.16%)	17 (3.60)	11 (15.49)	
1	54 (9.94%)	45 (9.53%)	9 (12.68%)	
2	365 (67.22%)	315 (66.74%)	50 (70.42%)	
3	96 (17.68%)	95 (20.13%)	1 (1.41%)	
General Health				<0.001
Bad	52 (9.58%)	45 (9.53%)	7 (9.86%)	
Fair	293 (53.96%)	256 (54.24%)	37 (52.11%)	
Good	178 (32.78%)	151 (31.99%)	27 (38.03%)	
Excellent to Very good	20 (3.68%)	20 (4.24%)	0 (0%)	
FEV_1%_				<0.001
≤ 35	45 (8.29%)	31 (6.57%)	14 (19.72%)	
36 to 50	140 (25.78%)	119 (25.21%)	21 (29.58%)	
51 to 64	217 (39.96%)	189 (40.04%)	28 (39.44%)	
≥ 65	141 (25.97%)	133 (28.18%)	8 (11.27%)	
BMI				0.02
≤ 21	28 (5.16%)	20 (4.24%)	8 (11.27%)	
>21	515 (94.84%)	452 (95.76%)	63 (88.73%)	
				

Data are presented as mean ± SD or number (%)

FEV_1% _= % FEV1 of predicted value.

Dyspnea from Fletcher ^12^

BMI: Body mass index.

Physical Activity: 0) 'doesn't leave the house, life is limited to the bed or armchair, or to doing some domestic chores', or 'leaves the house, but walks less than 100 meters'; 1) 'leaves the house and walks a few hundred meters, runs errands, but does not walk regularly'; 2) 'engages in physical activity in the vegetable garden', or 'takes walks for up to 8 kms, no less than five days a week'; and 3) 'takes walks regularly for longer than 8 kms, no less than five days a week', or 'practices sports'.

In the univariate analysis, both the BODE-index and the HADO-score were associated in their different categories with all-cause or respiratory mortality (Table 3).

**Table 3:** All-cause and respiratory mortality in the entire cohort

	All-Cause Mortality (n = 71)	*P*-value	Respiratory Mortality (n = 32)	*P*-value
BODE index		<.0001		<.0001
0 to 2 (260)	14 (5.4%)		5 (2%)	
3 to 4 (193)	26 (13.5%)		13 (7.2%)	
5 to 6 (71)	24 (33.8%)		9 (16.1%)	
7 to 10 (19)	7 (36.8%)		5 (41.7%)	
HADO score		<.0001		<.0001
0 to 4 (73)	23 (32.39%)		14 (43.75%)	
5 to 7 (265)	35 (49.30%)		11 (34.38%)	
8 to 12 (205)	13 (18.31%)		7 (21.88%)	
				

Data are presented as number (%)

For the BODE index, higher scores indicate greater severity of disease; for the HADO score, lower scores indicate greater severity of disease

To explore the impact of FEV_1 _on prognosis, we divided the sample into two groups, FEV_1 _≥ 50% and FEV_1 _< 50%. Among patients with FEV_1 _< 50%, the greater the severity of the BODE-index and the HADO-score, the higher the mortality. However, this trend was statistically significant only for the BODE- index. Among patients with FEV_1 _≥ 50%, a direct association was again seen between the severity of the BODE-index and HADO-score and both respiratory and all-cause mortality, but in this case the trend was statistically significant for both measures (Table 4).

**Table 4:** All-cause and respiratory mortality by BODE index and HADO score categories by FEV_1_%

	All-Cause Mortality	*P*-value	Respiratory Mortality	*P*-value
**FEV1 ≥ 50%**	(n = 36)		(n = 12)	
**BODE index**		<.0001		0.005
0 to 2 (256)	14 (5.5%)		5 (2%)	
3 to 4 (83)	12 (14.4%)		5 (6.6%)	
5 to 6 (19)	10 (52.6%)		2 (18.2%)	
7 to 10 (0)	0		0	
**HADO score**		<.0001		<.0001
0 to 4 (11)	7 (63.6%)		5 (55.6%)	
5 to 7 (157)	18 (11.5%)		2 (1.4%)	
8 to 12 (190)	11 (5.8%)		5 (2.7%)	
				
**FEV1 < 50%**	(n = 35)		(n = 20)	
**BODE index**		0.02		0.05
0 to 2 (4)	0		0	
3 to 4 (110)	14 (12.7%)		8 (7.7%)	
5 to 6 (62)	14 (22.6%)		7 (12.7%)	
8 to 10 (19)	7 (36.8%)		5 (29.4%)	
**HADO score**		0.23		0.39
0 to 4 (62)	16 (25.8%)		9 (16.4%)	
5 to 7 (108)	17 (15.7%)		9 (9%)	
8 to 12 (15)	2 (13.3%)		2 (13.3%)	
				

Data are presented as number (% of each category).

Note: For the BODE index, higher scores indicate greater severity of disease; for the HADO score, lower scores indicate greater severity of disease.

In the multivariate analysis of the total sample of 543 patients, patients with HADO scores reflecting moderate (score 5 to 7) or mild (score 8 to 12) severity of COPD had low all-cause mortality after adjustment by age, hospitalizations in the two previous years, and comorbidities. Among patients with moderate HADO scores, the odds ratio (OR) for all-cause mortality was 0.39 (95% confidence interval (CI), 0.21 to 0.73), and was even lower (OR, 0.20; 95% CI, 0.07 to 0.60) for those with mild HADO scores compared to the most severe HADO category. For respiratory mortality, a HADO-score reflecting mild COPD severity also predicted low mortality (OR, 0.18; 95% CI, 0.04 to 0.86). BODE-index scores reflecting mild COPD severity also predicted significantly lower all-cause mortality compared to the most severe category (score 7 to 10), while a moderate BODE-index (score 5 to 6) was not a statistically significant predictor of all-cause mortality (OR, 0.55; 95% CI, 0.17 to 1.73). For respiratory mortality, the multivariate analysis revealed statistically significant differences between all the categories compared with the most severe category. (Table 5)

**Table 5:** Multivariate logistic regression for mortality. Overall sample

	All cause mortality		Respiratory mortality	
	**OR (95% CI)**	***P*-value**	**OR (95% CI)**	***P*-value**

**HADO score**				
0 to 4	1		1	
5 to 7	0.43 (0.25, 0.87)	0.002	0.23 (0.09, 0.59)	0.002
8 to 12	0.25 (0.10, 0.58)	0.001	0.26 (0.09, 0.77)	0.02
**BODE index**				
0 to 2	0.12 (0.04, 0.38)	0.0004	0.05 (0.01, 0.23)	< 0.001
3 to 4	0.23 (0.08, 0.70)	0.01	0.13 (0.03, 0.50)	0.003
5 to 6	0.53 (0.17, 1.70)	0.29	0.20 (0.05, 0.87)	0.03
7 to 10	1		1	
				

Models were adjusted by age, previous hospital admissions and packs/year and Charlson Comorbidity Index.

Note: For the BODE index, higher scores indicate greater severity of disease; for the HADO score, lower scores indicate greater severity of disease

In the multivariate analysis that included only patients with FEV_1 _< 50%, HADO scores reflecting moderate and mild COPD severity were not significantly better predictors of all-cause or respiratory mortality compared with the reference category of severe HADO-score (0 to 4), while significant differences in all-cause and respiratory mortality were observed between patients with lower BODE- index scores (less severe disease) compared to those with the highest scores (most severe disease). Among patients with FEV_1 _≥ 50%, those with mild to moderate HADO scores had significantly lower all-cause and respiratory mortality than those with severe HADO scores. With the BODE-index, significant differences were observed only for all-cause mortality (Table 6).

**Table 6:** All-cause and respiratory mortality according to severity of COPD (FEV_1 _< 50% vs. FEV_1 _≥ 50%)

	FEV_1 _< 50%				FEV_1 _≥ 50%			
	**All-cause mortality**		**Respiratory mortality**		**All-cause ortality**		**Respiratory mortality**	
	**OR (95% CI)**	***P*-value**	**OR (95% CI)**	***P*-value**	**OR (95% CI)**	***P*-value**	**OR (95% CI)**	***P*-value**

**HADO score**								
Severe	1		1		1		1	
Mild to Moderate	0.83 (0.34, 1.98)	0.67	0.81 (0.28, 2.36)	0.70	0.03 (0.005, 0.16)	<0.001	0.001 (0.0003, 0.05)	< 0.001
**BODE index**								
Mild	0.24 (0.07, 0.82)	0.02	0.17 (0.04, 0.74)	0.02	0.11 (0.04, 0.37)	0.003	0.23 (0.03, 1.75)	0.16
Moderate	0.40 (0.11, 1.47)	0.17	0.23 (0.05, 1.11)	0.07	0.22 (0.07, 0.71)	0.01	0.50 (0.06, 3.95)	0.50
Severe	1		1		1		1	
								

Categories for HADO score: Severe (<5), Mild-Moderate (≥5); Categories for BODE store in patients with FEV < 50: Mild (≤4), Moderate (5 to 6), Severe (≥7). Levels for BODE score in patients with FEV >50; Mild (0 to 2); Moderate (3 to 4); Severe (>4). Models were adjusted by age, previous hospital admissions, packs/year and Charlson Comorbidity Index.

**Figure 1:**
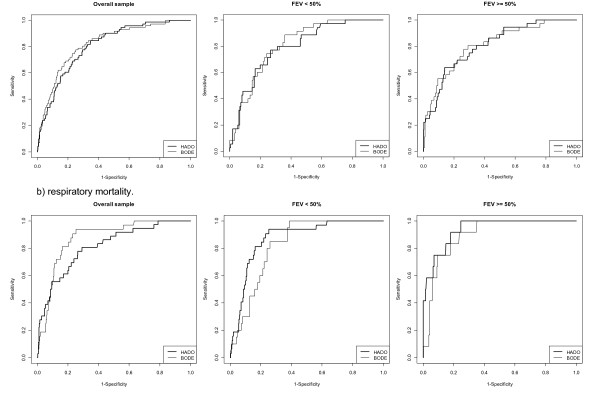
**ROC Curves for mortality**.

Figure 1 and Table 7 show the area under the curve (AUC) for the overall sample and both subsamples (FEV_1 _< 50% and FEV1 ≥ 50%), not being the BODE-index better than the HADO-score or vice versa.

**Table 7:** Comparison of the area under the curves for the mortality

	All-cause mortality			Respiratory mortality		
	**HADO score**	**BODE index**	***P*-value**	**HADO score**	**BODE index**	***P*-value**

**Overall sample**	0.81 (0.76, 0.86)	0.82 (0.77, 0.87)	0.17	0.86 (0.81, 0.91)	0.87 (0.82, 0.93)	0.56
**FEV_1 _< 50**	0.79 (0.72, 0.87)	0.81 (0.74, 0.88)	0.44	0.79 (0.69, 0.89)	0.83(0.76, 0.90)	0.29
**FEV1 ≥ 50**	0.81 (0.74, 0.88)	0.81 (0.74, 0.89)	0.83	0.94 (0.89, 0.99)	0.90 (0.84, 0.96)	0.06

## Discussion

This three-year study of 543 patients with COPD reinforces earlier findings that both the HADO-score and the BODE-index are good predictors of all-cause and respiratory mortality. However, differences appear between the two when applied to patients with different severity of COPD, as measured by FEV_1_%. In patients with the most severe disease (FEV_1 _< 50%), the BODE-index is a better predictor than the HADO-score of all-cause and respiratory mortality, whereas in patient with less severe disease (FEV_1 _≥ 50%), the HADO-score is as good predictor of respiratory mortality as the BODE-index.

The HADO-score and BODE-index arose from different patient populations. This is likely to determine their characteristics, qualities, and where each is most appropriately used. The BODE-index originated in a study of a sample of patients (recruited in hospitals), who are generally more seriously ill. In the studies defining these measures, almost 70% of the patients in the BODE-index cohort had an FEV_1 _< 50%, compared with 34% of those in the HADO-score cohort. In contrast, the HADO-score emerged from outpatient pneumology clinics, where the spectrum of COPD severity is wider, and generally lower, than it is among hospitalized patients. The HADO-score is also simpler to determine, which reflects the difficulty of conducting complex or time-consuming tests in the primary care setting.

Two variables shared by both measures are essential predictors of mortality among patients with COPD: FEV_1 _[1,2] and dyspnea. Although their importance has been questioned by some, it has been suggested that dyspnea is likely to be a better mortality predictor than FEV_1 _[3], though this has not been always replicated [22]. The different variables in the two measures are the patient's perception of his or her general health and self-reported physical activity in the HADO-score and BMI and the 6MWT in the BODE-index.

In our application of the HADO score, we introduced a single question to gauge a patient's sense of his or her level of health ('In general, would you say your health is...'). This question has proved to have good reproducibility, reliability, and strong concurrent and discriminatory scale performance with an established health status measure (SF-12) [23]. Moreover, it has been shown that this single health-related question can stratify patients with different risks for adverse outcomes such as mortality and the use of health resources [24]. A meta-analysis suggests a strong association between self-perceived health status and mortality, even after adjustment for key covariates such as functional status, depression, and co-morbidity, with individuals reporting *poor *health having higher mortality than their counterparts reporting *excellent *health (OR, 1.74; 95% CI, 1.51 to 2.02) [25].

The BODE-index includes BMI as a variable. It has been shown that BMI < 20 kg/m^2 ^constitutes an independent predictive factor related to COPD mortality [4]. In a study by Chailleux *et al*., the prevalence of malnutrition in patients with severe COPD who required home oxygen therapy ranged from 23% for men to 30% for women [26].The prevalence of malnutrition appears to be lower in the Mediterranean area. In a study of 3,126 patients, 50% of whom had a FEV_1 _< 50%, it was about 7% [27]. In the ambulatory setting, BMI offers limited prognostic value, since most patients seen in this setting have mild or moderate COPD, which generally does not cause malnutrition leading to BMI < 20 kg/m^2^. On the other hand, among patients with severe COPD, it is probably appropriate to carry out a precise measure of body composition, since it has been observed that lower fat-free mass is associated with higher mortality [28]. Since determining body composition would increase the complexity of obtaining the data for the prognostic index, its use should be reserved for patients with severe disease and should be exclusively handled in the hospital or specialized outpatient clinics.

The assessments of activity used in the two measures are also quite different. Self-reported physical activity, used in the HADO-score, is a behaviour (the activity the patient makes) that is quite simple to evaluate. The 6MWT, used in the BODE-index, reflects exercise capacity, a condition the patient reaches that allows him or her to meet the requirements of daily life (what the patient is able to do) [29]. The 6MWT is more difficult to measure. Both concepts, physical activity and exercise capacity, are indistinctly used in epidemiological studies.

Exercise capacity (as peak oxygen uptake, VO_2 _) has been associated with COPD mortality independent of FEV_1 _and age [6]. Among patients with severe COPD, the distance walked in six minutes has been shown to be an independent and better predictor of mortality than FEV_1 _[30]. In a recent study, the 6MWT had a stronger association with mortality (HR, 0.996; 95% CI, 0.993 to 0.999; *P *< 0.01) than did peak VO_2 _(HR, 0.971; 95% CI, 0.959 to 1.000; *P *= 0.050) [31].

Regarding physical activity, Garcia-Aymerich *et al*. showed in a population-based cohort that among patients with COPD, even slight physical activity (equivalent to walking or bicycling for two hours per week) had important consequences for the course of the disease, decreasing by 30% to 40% the risk of hospitalization due to COPD and the risk of respiratory mortality [7]. Interestingly, a questionnaire, or even a single question, on physical activity have proved to have acceptable associations with VO_2 _[32].

Despite the differences, both self-reported physical activity and exercise capacity appear to offer similar information concerning morbidity and mortality. Both are good predictors of mortality [33].

The differences in the HADO-score and BODE-index as predictors of mortality among patients with FEV_1 _< 50% is worth noting. Although both appear to behave similarly when analyzed using ROC curves (Figure 1), the multivariate analysis yields several differences. This could be due to the cut-points chosen, or because the HADO-score has only three categories of severity, compared to four for the BODE-index.

One limitation of our study is that the sample was composed almost entirely of men, which makes it impossible to generalize the results to women as well. This reflects the fact that in Spain, smoking was once for men only, and women began smoking relatively recently. It is possible that the use of self-reported physical activity in creating the HADO-score could influence the results, since we did not objectively measure the level of activity by accelerometers. Given the size of the cohort, this would have been a difficult undertaking. The main goal of this work was to study the functioning of both scores (BODE and HADO) in patients with different severity. Apart from using multidimensional scores, which is the main point of this work, there is not any other alternative form of classifying severity of COPD than using FEV_1_. Nevertheless, this choice has some disadvantages since this division of our sample (FEV1 < 50% vs FEV_1 _≥ 50%) reduces the number of deaths in each category and thus the power of the study to detect differences between the BODE and the HADO in their ability to predict mortality. But besides that, we were able to detect differences.

Strengths of this study are the prospective collection of data, up to three years of follow-up of a relatively large cohort of COPD patients covering a wide range of severity of the disease and the evaluation of the utility of two COPD-specific severity scales in relation to robust outcomes, such as mortality.

## Conclusion

Both the HADO-score and the BODE-index are supported by variables that have been shown to be consistent predictors of mortality among patients with COPD. In general, the two measures generally perform similarly. However, the BODE-index yields better results among patients with the most severe COPD (FEV_1 _< 50%), who are likely to be assessed at the hospital level. Among patients with less severe disease (FEV_1 _≥ 50%), who comprise the majority of patients with COPD and who are generally seen in the outpatient or primary care setting, the HADO-score is as good a predictor of respiratory mortality, a crucial parameter in these patients, as the BODE-index. The HADO-score is also easier to compile in the outpatient and primary care settings. Therefore, we propose that these instruments are appropriate for different settings. The HADO-score offers a quick, simple assessment suitable for use by practitioners seeing patients with mild to moderate COPD and who do not immediately require more thorough evaluation. The BODE-index is more suitable for the evaluation of patients with more severe COPD, who require more accurate and specialized assessment requiring more resources of time and personnel. That said, the use of both indexes is not incompatible and they could even be complementary in the different care levels.

## Abbreviations

6MWT: 6-minute walking test; ATS: American Thoracic Society; AUC: area under the curve; BMI: body mass index; BODE-index: Body mass index, Obstruction, Dyspnea, Exercise capacity-index; COPD: Chronic Obstructive Pulmonary Disease; FEV_1_: forced expiratory volume in one second; FVC: Forced Vital Capacity; HADO-score: Health, Activity, Dyspnea, Obstruction-score; OR: odds ratio; SEPAR: Spanish Pneumology and Thoracic Surgery Society.

## Competing interests

The authors declare that they have no competing interests.

## Authors' contributions

CE, JMQ, MA, FJM and SA participated in the design and implementation of the study. MA, FJM, JIA and AC provided critical contributions to the drafts of the manuscript. CE, JMQ, UA, SA and LVB collected and analysed the data. CE and SA supervised the data collection. CE and JMQ wrote the first and all consecutive drafts of the manuscript. All authors read and approved the final manuscript.

## Pre-publication history

The pre-publication history for this paper can be accessed here:

http://www.biomedcentral.com/1741-7015/8/28/prepub
